# Effectiveness of a Care Delivery Model for High-Need Community-Dwelling Older Adults

**DOI:** 10.1093/geroni/igaa057.893

**Published:** 2020-12-16

**Authors:** Kuei-Min Chen, Hui-Fen Hsu

**Affiliations:** 1 Kaohsiung Medical University, Kaohsiung City, Taiwan (Republic of China); 2 Kaohsiung Medical University, Kaohsiung, Taiwan (Republic of China)

## Abstract

The effectiveness of sufficient care coordination for high-need community-dwelling older adults has not been discussed. This study aimed to examine the effectiveness of a newly-developed care delivery model for high-need community-dwelling older adults. A cluster randomized controlled trial with repeated measures design was employed. A total of 145 high-need older adults participated in the study and were randomly assigned to either the intervention group or comparison group. A categorized list of care services based on the types of high-need older adults as the intervention allowed care coordinators to make adequate care service linkages. The intervention period ranged over 6 months with regulated home visits and assesssments. Functional ability, quality of life, depressive symptoms, and healthcare and social service utilizations were measured at baseline, and at 3 and 6 months into the intervention. The participants’ satisfaction with care delivery was measured at the end of 6-month intervention. Results showed that the intervention group had a better functional ability, a higher quality of life, reduced depressive symptoms, and more efficient healthcare and social service utilizations than the comparison group at both the 3-month and 6-month intervals (all p < .05). By the end of the 6-month study, the intervention group were more satisfied with the care service linkages than the comparison group (p < .05). The positive effects of providing a categorized list of care services for care coordinators to make service linkages have been evidenced by the outcomes. The promising findings supported a further longer-term implementation of the care delivery model.

